# Toward Sustainable Aquaculture: Microalgae Fully Replace Fishmeal and Fish Oil in Rainbow Trout Diets While Maintaining Growth, Nutritional Quality, and Cost Viability

**DOI:** 10.3390/antiox15101343

**Published:** 2026-10-16

**Authors:** Pallab Sarker, Manuel Labbe, Benjamin V. Schoffstall, Anne R. Kapuscinski, Brandi McKuin, Devin Fitzgerald, Connor Greenwood, Kira O’Shelski, Emily Noelle Pasion, Duncan Gwynne, Diego Gonzalez Orcajo

**Affiliations:** 1 Environmental Studies Department, University of California Santa Cruz, CA 95064, USA; manny.labbe@gmail.com (M.L.); bschoffs@ucsc.edu (B.V.S.); akapusci@ucsc.edu (A.R.K.); kiraoshelski@gmail.com (K.O.); dgwynne@ucsc.edu (D.G.); dgonz@ucsc.edu (D.G.O.); 2 School of Engineering, University of California, 5200 Lake Rd, Merced, CA 95340, USA; bmckuin@ucmerced.edu; 3 California Sea Grant Extension Program, University of California, San Diego, CA 92093, USA; defitzgerald@ucsd.edu; 4 Monterey Bay Salmon and Trout Project, Big Creek Rd, Davenport, CA 95017, USA; cgreenwood@mbstp.org; 5 Keck Graduate Institute in Claremont, 535 Watson Dr, Claremont, CA 91711, USA; ecpasion@ucsc.edu

**Keywords:** aquaculture feed, microalgae *Nannochloropsis oculata* by-product, *Schizochytrium* sp., ocean-derived fishmeal and fish oil, sustainability, rainbow trout, growth, nutritional quality, economic viability

## Abstract

As global demand for affordable, high-quality protein continues to rise, commercial aquaculture has emerged as a critical solution. The industry’s reliance on ocean-derived fishmeal (FM) and fish oil (FO) for aquafeed poses sustainability concerns. Marine microalgae offer a promising alternative due to their comparable nutrient profiles and potential for large-scale, sustainable production. We conducted a feeding trial with juvenile rainbow trout to evaluate fish-free, microalgae-based diets as alternatives to FM and FO. Diets were formulated using *Nannochloropsis oculata* (*N. oculata*) by-product as a protein source and *Schizochytrium* sp. (whole-cell or oil) as a source of DHA and key antioxidants. Diets included a reference formulation containing FMFO and three experimental diets: 75% replacement of FMFO using *N. oculata* and *Schizochytrium* whole-cell (NSW75), 100% replacement using both (NSW100), and 100% replacement using *N. oculata* and *Schizochytrium* oil (NSO100). The NSW100 diet supported growth and feed conversion comparable to the FMFO control; the NSO100 had lower growth than the FMFO control. Cost analysis revealed that fish-free diets had the numerically lowest, though not significantly different, values for formulated feed costs and economic conversion ratios, highlighting their commercial potential. These findings demonstrate that microalgae-based aquafeeds combining *N. oculata* and *Schizochytrium* sp. can viably replace FMFO.

## 1. Introduction

Aquaculture has rapidly become a key pillar of global seafood production, surpassing capture fisheries for the first time in 2022 with 94 million tons of output [1]. By 2030, over 60% of seafood is projected to come from aquaculture, making it essential to food security, nutrition, and economic development [2,3]. This growth is driven by rising population, incomes, health-conscious diets, and declining wild fish stocks [4,5]. However, aquaculture’s reliance on fishmeal (FM) and fish oil (FO), primarily derived from wild-caught marine species such as sardines, anchovies, herring, and mackerel, poses a significant sustainability challenge [6,7]. Each year, approximately 16 million tons of wild forage fish—largely edible—are converted into FM and FO, with aquaculture consuming 87% of global FM and 74% of FO [2,8]. This dependence contributes to overfishing, disrupts marine ecosystems, exacerbates food insecurity in low-income regions, and exposes the industry to environmental and economic instability due to resource scarcity, climate change, and price volatility [5,7,9,10]. The urgent need for sustainable and cost-effective alternatives has driven extensive research in aquaculture [5,7,11,12].

Replacing FM and FO is especially critical for carnivorous species like rainbow trout, which require high levels of digestible protein and omega-3 fatty acids. Terrestrial plant-based alternatives such as soy, corn, and canola oil have become more commonplace, but require large-scale land use, contributing to land-use deforestation [13], freshwater pollution from fertilizer runoff [12,14], and other issues caused by industrial farming [15]. Furthermore, terrestrial crops contain anti-nutritional factors and lack essential amino acids, n-3 LC-PUFA, and cholesterol, resulting in compromised growth and health in farmed salmonids [16,17,18,19,20,21].

A promising solution lies in microalgae, which can be cultivated sustainably and offer high-quality protein, amino acids, omega-3s, and antioxidants [22,23]. Marine microalgae are proving to be more viable replacements for FM and FO as they contain good amino acid and fatty acid profiles [19,21,24,25,26,27,28,29]. Marine microalgae also have many bioactive compounds and have been shown to have favorable antioxidant profiles for aquatic species [30,31]. These bioactive compounds have been shown to improve antioxidant capacity and immune functioning in rainbow trout [23,30,32]. Current global production of autotrophic microalgal biomass remains relatively modest, at approximately 20,000 tons (dry weight) [33]. However, demand for high-value microalgal-derived products continues to expand rapidly, with the global EPA/DHA market reaching approximately 124 thousand tonnes and valued at nearly €2 billion [34]. This growing demand highlights both the opportunity and necessity to diversify microalgal resources for aquafeed applications. Expanding the portfolio of microalgal raw materials through continued innovation can enhance flexibility in feed formulation while improving nutritional optimization, ingredient availability, sustainability, and cost-effectiveness [19,35]. Microalgae are also becoming more readily available for use in aquafeed; as nutraceutical and biofuel industries grow, companies seek markets for the leftover microalgal by-product. 

Recent research has demonstrated that the residual microalgal by-product from *Nannochloropsis* sp. biomass, generated after extraction of EPA-rich oil for human nutritional supplements, can support healthy growth in rainbow trout without the inclusion of FM [19]. This by-product currently has little or no commercial use, has no established market price, and therefore represents an underutilized resource for aquaculture feed. *Nannochloropsis* is cultivated for producing high-value oils rich in EPA for human nutraceutical and functional food markets, leaving residual biomass streams that may represent a promising and economically relevant feed ingredient for aquaculture [18,25].

The replacement of FM and FO with alternative ingredients will be critical in advancing a more sustainable and stable future for aquaculture. This study aims to take advantage of available underutilized microalgal by-products and formulate economically viable, fish-free feeds for rainbow trout. We designed a feeding experiment comparing three microalgal diets to a reference diet containing FM and FO in commercial rainbow trout feed. We measured the effects of the four diets on growth, feed conversion ratio (FCR), protein efficiency ratio (PER), and whole-body and fillet deposition of macronutrients, amino acids, and n-3 long-chain polyunsaturated fatty acids (PUFAs). Furthermore, we estimated the formulated feed cost and the economic conversion ratio (ECR) for the three different experimental diets and the reference diet.

## 2. Materials and Methods

An 84-day growth experiment was designed to replace fish meal with *N. oculata* by-product and to replace fish oil with *Schizochytrium* sp. whole cells and oil. All experimental procedures involving fish were conducted at the Center for Agroecology, University of California, Santa Cruz, in accordance with institutional guidelines. The experimental design and fish use protocol were approved by the Institutional Animal Care and Use Committee (IACUC approval number: Sarkp2302dn_r2).

### 2.1. Diet Formulation and Nutritional Feeding Experiment

To meet the complete nutritional requirements of juvenile rainbow trout, the dietary treatments were formulated to be iso-nitrogenous, iso-energetic, and iso-lipidic, ensuring consistency in protein, energy, and lipid content across treatments (Table 1) [19,27]. The diet’s nutritional composition based on this formulation strategy is presented in Table 2. The reference diet (FMFO) contained fishmeal (FM) and fish oil (FO). Three experimental diets used *N. oculata* by-product as the primary protein source and either *Schizochytrium* sp. whole cells or a combination of *Schizochytrium* oil and canola oil as sources of oil. The three experimental diets are described as follows: NSW75 diet replaced 75% of the FM with *N. oculata* by-product meal (6%) and 75% of the FO with a blend of *Schizochytrium* whole cells (5%) and canola oil (9%), NSW100 diet completely replaced FM with *N. oculata* by-product meal (8%) and FO with a blend of *Schizochytrium* whole cells (5%) and canola oil (12%), and NSO100 diet completely replaced FM with *N. oculata* by-product meal (8%) and FO with a blend of *Schizochytrium* oil (2.8%) and canola oil (12%). The experimental diets were designed to evaluate practical fishmeal- and fish oil-replacement strategies using commercially relevant formulations rather than isolate the effects of individual ingredients. Accordingly, comparisons between the reference (FMFO) diet and the replacement diets, as well as between the 75% (NSW75) and 100% (NSW100) replacement diets, represent the combined effects of simultaneous changes in both protein and lipid sources. In contrast, the NSW100 and NSO100 diets were formulated identically except for the form of the *Schizochytrium* ingredient (whole-cell biomass versus extracted oil), while all major ingredients and inclusion levels were held constant. This pairwise comparison therefore directly assesses the effect of *Schizochytrium* form, independent of other dietary variables.

Our formulation strategy prioritized developing iso-nitrogenous, iso-lipidic, and iso-caloric experimental diets relative to the FMFO reference diet so that fish performance could be evaluated without confounding effects of dietary protein, lipid, or energy. Limiting amino acids (methionine and lysine) and taurine were supplemented equally across all diets (Table 1). All diets were formulated to meet or exceed the established essential amino acid requirements of rainbow trout [36]. Because the *N. oculata* by-product contained lower crude protein and essential amino acid concentrations than fishmeal, its inclusion level was adjusted during formulation to maintain iso-nitrogenous diets. To further ensure nutritional adequacy and minimize amino acid deficiency as a confounding factor, the first-limiting amino acids, methionine and lysine, were supplemented equally across all dietary treatments. Although the experimental diets were not formulated to replicate the fatty acid profile of the reference diet, they were designed to meet or exceed the established essential fatty acid requirements for rainbow trout recommended by [36], including α-linolenic acid (18:3n-3; 0.7–1.0% of diet), linoleic acid (18:2n-6; approximately 1.0% of diet), and long-chain n-3 PUFA (EPA + DHA; 0.4–0.5% of diet) (Table S1. Fatty acid profiles of experimental diets). Lipids were supplied primarily by whole-cell biomass or oil from the DHA-rich microalga *Schizochytrium* sp., supplemented with canola oil to provide energy and α-linolenic acid while replacing fish oil. Consequently, the experimental diets differed from the FMFO reference diet in their overall fatty acid profiles, particularly in EPA and DHA concentrations, reflecting the inherent composition of the alternative lipid sources rather than an attempt to duplicate marine ingredient fatty acid profiles. Table S2 shows the micromineral and trace element content of the experimental diets.

Table 2 presents the proximate composition, amino acid profiles, and antioxidant content of the diets, while Supplementary Tables S1 and S2 report their fatty acid composition and mineral content, respectively.

*Schizochytrium* sp. was purchased from Algamac™, Aquafauna Bio-marine, Inc., Hawthorne, CA, USA; and menhaden FO from Double Liquid Feed Service, Inc., Danville, IL, USA. Qualitas Health Inc., Houston, USA, which markets EPA-rich oil extracted from *N. oculata* as a human supplement, donated the lipid-extracted *N. oculata* by-product generated from its large-scale production facilities. Although a substantial portion of the oil is extracted, the by-product retains residual lipids, including EPA (27.94% of total fatty acids; Table 3), highlighting its nutritional value as a sustainable aquafeed ingredient. *Schizochytrium* sp. DHA oil was purchased from Algarithm (Bay of Fundy, Nova Scotia, Canada). Table 3 reports proximate compositions, amino acid profiles, and antioxidant content (Carotene, Xanthophyll, Vitamin E) of *N. oculata* by-product, *Schizochytrium* sp., fish meal, and fish oil. Table S3 reports the total fatty acid profile by percentage of the by-product, *Schizochytrium* sp. whole cells ingredient, and *Schizochytrium* sp. oil; and Table S4 reports the macromineral and trace element composition of the ingredients.

Each diet contained yttrium oxide (Y_2_O_3_), a digestibility marker sourced from Thermo Scientific, Waltham, MA, USA, in the basal diet at a rate of 1.0% [38]. The digestibility marker was included in all experimental diets because the primary objective was to determine and compare the apparent protein digestibility coefficients among dietary treatments using a consistent formulation, and no separate diets without the marker were prepared. To manufacture the experimental diets, micro-ingredients were first thoroughly mixed, followed by gradual addition and mixing of macro-ingredients to produce a homogeneous mash. Diets were manufactured at the Kapuscinski–Sarker Laboratory (Natural Sciences II, University of California, Santa Cruz, CA, USA) using a TT-100 single-screw laboratory extruder (Akron Tool and Die, Akron, OH, USA). The feed mash was extruded at an average barrel temperature of 90 °C with an approximate residence time of 18 s. This relatively low extrusion temperature was intentionally selected to minimize thermal degradation of functional microalgal ingredients and essential nutrients, as our previous study showed that higher extrusion temperatures (approximately 127 °C) can reduce nutrient quality, including methionine availability [38]. Following extrusion, the appropriate pellets were top-coated with fish oil, *Schizochytrium* whole-cell, or *Schizochytrium* oil and canola oil using a rotating mixer (SUNCOO 4/5 HP Electric Concrete Mixer, SUNCOO) for 15 min. to ensure uniform coating. The coated pellets were then dried overnight in a fume hood to reduce moisture to approximately 16–18%, thereby improving pellet stability for handling and storage. After drying, the pellets were sieved and stored at −20 °C until use. Fish were initially fed 2.0-mm pellets, and pellet size was increased to 4.0 mm as the fish grew to accommodate their larger mouth size.

### 2.2. Fish Husbandry and Experimental Design

Sixteen replicate recirculating aquaculture systems at the University of California, Santa Cruz, CA, USA, were used, each one with independent water recycling for its 757 L tank, and containing 40 juvenile rainbow trout with an average weight of 0.85 g. Juvenile rainbow trout (*Oncorhynchus mykiss*) were obtained from Thomas Fish Company (Anderson, CA, USA) and acclimated to laboratory conditions prior to the feeding trial. Following placement in tanks, the fish were allowed to acclimatize for seven days. During this period, all tanks received the same commercial rainbow trout diet to apparent satiation twice daily to standardize nutritional status before the feeding trial and minimize behavioral variation, including aggressive interactions and fin biting. After the acclimation period, we randomly assigned the three experimental diets and reference diet to the 16 tanks, with four replicate tanks per diet, and fed fish until apparent satiation twice a day, in the morning and afternoon, six days a week, for 84 days. Each tank was monitored to maintain recommended conditions for rainbow trout [19,38]. Dissolved oxygen, dissolved oxygen saturation, temperature, and pH were measured daily using a handheld YSI Pro1020 multiparameter, YSI, Yellow Springs, USA, water meter. Dissolved oxygen levels were maintained at or above 8.7 mg/L by adjusting aeration and water flow rates as needed. Water temperature was controlled to remain below 15.4 °C, and pH was maintained below 8.6. Ammonia, nitrite, nitrate, and alkalinity of the water were measured weekly using a benchtop YSI 9500 spectrophotometer to maintain total ammonia nitrogen at or below 0.2 mg/L, nitrite nitrogen at or below 0.1 mg/L, and nitrate nitrogen at or below 26.8 mg/L.

Fecal samples were collected daily prior to feeding using a modified settlement column approach based on the Guelph system [39], which is widely recognized for minimizing nutrient leaching. A radial flow settler was installed between the culture tank outflow and sump tank inflow to enhance fecal recovery efficiency and facilitate the collection of intact fecal material at the bottom of the system [38]. Uneaten feed pellets were carefully removed by siphoning to prevent contamination. Intact fecal matter was gently collected from a dedicated collection bin using pipettes, transferred into 50 mL Falcon tubes (BD Falcon™, Corning, Corning, USA), and allowed to settle, after which the supernatant was removed with minimal disturbance. To further minimize nutrient degradation and leaching, samples were retrieved immediately after excretion following standardized digestibility protocols [19,38,40]. Fecal material was processed promptly and frozen within a short time frame at −20 °C (or lower), and repeated freeze–thaw cycles were avoided to preserve sample integrity. The reliability of this approach for rainbow trout has been validated in previous studies [27,38].

Fecal samples were pooled by tank throughout the experimental period. At the conclusion of the trial, samples were lyophilized, finely ground, and stored at −20 °C until nutrient analysis.

At sampling, the total fish biomass in each tank was measured. From each tank, three fish were euthanized for whole-body proximate and biochemical composition analysis, while an additional three fish were filleted using a standardized dorso-anterior landmark. Fillet samples were individually packaged in sterile polyethylene bags (Whirl-Pak^®^, Filtration Group, Austin, USA) and stored at −20 °C until subsequent analysis.

### 2.3. Growth Calculations

Growth, Weight gain (%), feed intake, FCR, SGR, PER, and survival rate were quantified for each dietary treatment. These parameters were calculated as follows:

Weight (Wt.) gain (g) = final Wt. − initial Wt.(1)

Wt. gain (%) = (final Wt. − initial Wt.)/initial Wt. × 100.(2)

Feed Intake (g) = diet intake/fish(3)

Feed conversion ratio (FCR) = feed intake (g)/Wt. gain (g).(4)

Specific growth rate (SGR) (%/day) = 100% × (ln final wet Wt. (g) − ln initial wet Wt. (g))/Time (days).(5)

Protein efficiency ratio (PER) = Wt. gain (g)/protein fed (g).(6)

Survival (%) = (Final number of fish/Initial number of fish) × 100%.(7)

### 2.4. Macronutrient, Antioxidants, Trace Element Analysis and Digestibility Calculations

All experimental materials, including microalgal ingredients, experimental diets, fecal samples, whole-body, and fillet samples, were submitted to New Jersey Feed Laboratory, Inc. (Ewing, NJ, USA) for comprehensive compositional analysis [41]. Analyses included moisture (AOAC 930.15), crude protein (AOAC 990.03), crude lipid (AOAC 920.39), ash (AOAC 942.05), crude fiber (AOAC 978.10), and gross energy (determined using an automated oxygen bomb calorimeter). Amino acid profiles were quantified using high-performance liquid chromatography (HPLC) according to AOAC methods 994.12, 985.28, 988.15, and 994.12. Fatty acid composition was determined as fatty acid methyl esters (FAME) following AOAC method 963.22.

Samples of the microalgal ingredients, fishmeal, fish oil, and experimental diets were submitted to New Jersey Feed Laboratory, Inc. (Ewing, NJ, USA) for analysis of antioxidants (carotene, xanthophylls, and vitamin E). Carotene and xanthophyll concentrations were determined according to AOAC 970.64 using solvent extraction followed by chromatographic separation and spectrophotometric quantification. Vitamin E was analyzed according to AOAC 988.14/989.09/969.40 (modified), involving saponification and solvent extraction followed by gas chromatographic (GC) determination. These analyses were used to characterize the antioxidant-related composition of the feed ingredients and experimental diets and to assess their potential contribution to the functional properties of the microalgae-based formulations.

Nutrient composition was determined after homogenously grinding and freezing ingredients, diets, feces, and whole-body fish at −20 °C. Samples were prepared for ICP-OES analysis using the EPA Method 3050b for acid digestion [42], and the digested material was analyzed for elemental composition by ICP-OES (Thermo iCap 7400 radial view ICP-OES, Optical Emission Spectroscopy, Thermo Fisher Scientific, Waltham, USA) at the UC Santa Cruz Plasma Analytical Laboratory (RRID:SCR_021925).

After determining the concentrations of crude protein and yttrium in the diets and feces, the apparent digestibility coefficient (ADC) of protein was calculated.

The following equation was used to determine the ADC for the four diets [39]:

ADC = (1 − ((%Protein feces/%Protein feed)/(%Y2O3 feed/%Y2O3 feces))) × 100)(8)

### 2.5. Economic Conversion Ratio

The Cruz Aquafeed Sustainability Tool (CAST; https://cast.sites.ucsc.edu/, accessed on 1 November 2025) was used to calculate the economic conversion ratio (ECR), employing market prices from the CAST database to estimate ingredient costs [19,43]. In CAST, we selected the option to apply the experimental feed conversion ratios (FCRs) obtained in this study rather than the default algorithm-generated values. The market prices of the experimental diets from the CAST database were obtained from a variety of sources (Table S5). Although the economic analysis was conducted using the Cruz Aquafeed Sustainability Tool (CAST) (v1) in 2025, the ingredient market prices used were obtained from the CAST database and represent market values compiled from multiple sources between 2020 and 2022, corresponding to the period during which the experimental diets were formulated and the feeding trial was conducted (Table S5). These prices were therefore used to estimate the economic conversion ratio under market conditions representative of the experimental period. The median values and 95% confidence intervals of market prices in CAST were estimated using non-parametric bootstrapping in RStudio (v.1.2.5033), based on 10,000 replicates and applying the adjusted bootstrap percentile method. The fish production cost was then expressed as the ECR [27]:

ECR ($/kg fish) = FCR ((kg diet fed)/(kg weight gain)) × price of diet (USD/kg diet)(9)

where ECR represents the economic conversion ratio, and FCR denotes the feed conversion ratio.

### 2.6. Statistical Analysis

Significance among treatment groups was determined using one-way ANOVA in IBM SPSS Statistics Version 27. All assumptions for ANOVA were tested using Levene’s test for homogeneity and Shapiro–Wilk’s test for normality. If there was a significant difference (*p* < 0.05) between treatment groups, we included a Tukey post hoc test in determining the similarities and differences between treatments when *p* < 0.05.

## 3. Results

The experiment was conducted to determine the effects of replacing a reference diet of FM and FO with different percentages of *N. oculata* to replace FM, and either whole-cell *Schizochytrium* sp. or *Schizochytrium* oil to replace FO.

### 3.1. Growth and Feed Performance

Growth and feed performance indices for the different dietary treatment groups are presented in Table 4. Rainbow trout fed experimental diets had a statistically similar feed conversion ratio (FCR) to the trout fed the reference diet of FMFO (*p* > 0.05). Apparent digestibility coefficient (ADC) of crude protein was significantly higher (*p* < 0.05) in the NSW100 and NSO100 treatment groups compared to the NSW75 and reference groups (Table S6).

Weight gain and final weight did not significantly differ (*p* > 0.05) between the reference group, NSW75 (where FMFO were partially replaced), and NSW100 (where FMFO were fully replaced with *N. oculata* and *Schizochytrium* sp. whole cells). However, weight gain and final weight were significantly higher (*p* < 0.05) in the reference group compared to the NSO100 group. This result emphasizes the important differences in DHA-extracted *Schizochytrium* sp. oil rather than *Schizochytrium* sp. whole cells in replacing FO. Final weight ranged from 32.9 g to 35.0 g per fish between groups.

Trout appeared healthy at the end of the experiment with no signs of illness or deformities, and there was no difference in survival between the dietary treatment groups.

### 3.2. Fish Whole-Body Proximate Composition and Fatty Acid Content

We compared the whole-body proximate composition across all dietary treatment groups (Table 5). Trout fed the fish-free diets (NSW100 and NSO100) did not differ significantly in moisture content from either the reference (FMFO) or the partial-replacement (NSW75) diets. Trout fed fish-free diets (NSW100, NSO100) had similar whole-body protein as those fed the reference diet, but significantly more protein than the partial-replacement diet (NSW75). The protein content ranged from 48.36–53.06%; numerically highest in the NSW100 group where FMFO was fully replaced. Fat content ranged from 34.29 to 36.87%; highest in the NSW75 group where FMFO was partially replaced. The fiber content range was 1.34–2.01%, and ash content range was 4.78–5.22%.

The fatty acid profile of whole-body fish is presented in Table 6. The fatty acid content of diets differed and is presented in Table S1. Trout fed the reference diet had significantly higher (*p* < 0.05) saturated fatty acids (SFA), but significantly lower (*p* < 0.05) monounsaturated fatty acids (MUFA) compared to trout fed the experimental diets. For SFA, the reference group had significantly higher (*p* < 0.05) myristic (14:0), pentadecanoic (15:0), palmitic (16:0), heptadecanoic (17:0), stearic acid (18:0), and significantly lower arachidic acid (20:0) compared to treatment groups. Between treatment groups, NSW100 had the highest numeric arachidic acid (20:0) content, and NSO100 had significantly lower heptadecanoic acid (17:0) compared to the reference and NSW75. For MUFA, the reference group had significantly higher (*p* < 0.05) palmitoleic (16:1n9), palmitoleic (16:1n7), and oleic (18:1n7), and significantly lower (*p* < 0.05) oleic (18:1n9), eicosanoic (20:1n9), and erucic (22:1n9) content compared to the treatment groups. Between treatment groups, NSW75 had significantly higher (*p* < 0.05) palmitoleic (16:1n7) than NSW100 and NSO100, significantly lower (*p* < 0.05) eicosanoic acid (20:1n9) compared to the NSW100, and significantly lower total MUFA compared to NSW100 and NSO100 diets. SFA ranged from 25.84% of TFA for NSO100 to 47.98% of TFA for the reference diet. MUFA ranged from 45.39% of TFA for the reference diet to 65.77% of TFA for NSW100.

Total polyunsaturated fatty acids (PUFA) content was similar across groups. We found no significant difference in total n-6 PUFA (Omega-6), including arachidonic acid (ARA, 20:4n6), linoleic acid (LA,18:2n6), and total n-6 LC-PUFA content; each ranged from lowest in the reference group to highest in the NSO100 group (Table S7). Similarly, we found no significant difference in total n-3 PUFA (Omega-3) between groups, including no significant difference in alpha-linolenic acid (ALA, 18:3n3), docosahexaenoic acid (DHA, 22:6n3), and total n-3 LC-PUFA. However, we detected significantly higher (*p* < 0.05) eicosapentaenoic acid (EPA) content in the reference group compared to the experimental groups. The n-3/n-6 PUFA ratio (n-3/n-6) was significantly higher (*p* < 0.05) in the reference group compared to the experimental groups.

### 3.3. Fillet Proximate and Amino Acid Profile

We compared the fillet proximate composition across all dietary treatment groups (Table 7). We found no significant difference (*p* > 0.05) in moisture, protein, fat, fiber, or ash between groups. The lipid content ranged from 14.30% to 20.37% across groups and was highest in the NSO100 group. The protein content ranged from 69.70% to 75.85% across groups; numerically highest in the NSW100 group.

The amino acid content was similar across groups (Table 8). The fillet of the reference group had slightly lower levels of methionine, phenylalanine, leucine, threonine, and histidine when compared to the experimental groups. The content of methionine ranged from 1.85% to 2.00%; phenylalanine from 2.62% to 2.80%; leucine from 4.98% to 5.14%; threonine from 3.05% to 3.11%; histidine from 2.02% to 2.13%.

### 3.4. Whole-Body Macro Minerals and Trace Element Content

We did not find any significant differences in the macro mineral composition of the whole-body (Table 9). The phosphorus composition ranged from 0.99% to 1.11%; calcium from 0.59% to 0.85%; magnesium from 0.08% to 0.09%; potassium from 1.04% to 1.13%; and sulfur from 0.63% to 0.69%.

We found no significant difference in the trace element composition of the whole body. We found a higher percentage of copper in the reference diet (Table S2), and a lower percentage of iron compared to the experimental groups, though no significance was found. Iron was highest in the NSW75 group, ranging from 33.6 to 47.4 mg/kg. Zinc was highest in NSW100, ranging from 34.39 to 71.55 mg/kg. Amounts of selenium, arsenic, and lead were below the level of detection of the instrument.

### 3.5. Antioxidant Content of Diets and Ingredients

All diets had similar levels of xanthophyll (2.6–3.9 mg/kg) and vitamin E (193–230 IU/kg). The NSO100 diet had numerically lower carotene (0.4 mg/kg) than all other diets (1.1–1.7 mg/kg) (Table 2). The *Nannochloropsis* by-product, *Schizochytrium* sp. whole cell, *Schizochytrium* sp. oil, and fish meal showed varying levels of carotene (9.0 mg/kg, 23.2 mg/kg, 0.2 mg/kg, and 1.1 mg/kg) and vitamin E (31 IU/kg, <25 IU/kg, 1700 IU/kg, and <25 IU/kg), respectively. Xanthophyll was higher in *Schizochytrium* sp. whole cell (5.2 mg/kg) than in *Schizochytrium* sp. oil (0.3 mg/kg) (Table 3).

### 3.6. Economic Conversion Ratio

As shown in Table 10, the feed conversion ratio (FCR) and economic conversion ratio (ECR) did not differ significantly (*p* > 0.05) between diets. NSW100 had both the lowest ECR and formulated feed cost (FFC); ECR ranged from 0.77 to 0.86 USD per kg of trout, and FFC ranged from 0.87 to 0.97 USD per kg of feed. NSO100 had the highest (worst) feed conversion ratio; feed conversion ranged from 0.88 to 0.98 g feed intake per gram wt. gain. Market prices for the experimental diets were obtained from the CAST database, compiled from a variety of sources (Table S5).

## 4. Discussion

This study provides the first evidence that rainbow trout can achieve comparable growth, FCR, and survival when fed a fully fish-free diet in which microalgal biomass replaces 100% of both FM and FO than when fed the FMFO reference diet—while remaining cost-viable. A blend of *Nannochloropsis oculata* by-product and DHA-rich *Schizochytrium* sp. whole cells served as effective replacements for FM and FO, with no loss in performance and a comparable economic conversion ratio (ECR). Moreover, the 100% fish-free diets showed higher protein digestibility than the FMFO reference diet. These results underscore the potential of sustainable microalgal ingredients as viable alternatives in aquaculture, with promising implications for both environmental sustainability and economic feasibility.

### 4.1. Growth Performance and Apparent Digestibility

While prior studies have successfully replaced either FM or FO individually, full replacement of both FM and FO has frequently resulted in compromised performance due to low feed palatability, poor digestibility, or nutrient deficiencies [18,44,45,46,47,48]. Our initial attempt to formulate fish-free feed for rainbow trout using a microalgal blend was unsuccessful, primarily due to reduced feed intake and poor palatability [27].

In this study, in contrast, the NSW75 and NSW100 diets, combining *N. oculata* and *Schizochytrium* sp. whole-cell biomass, supported equivalent growth, feed conversion ratio (FCR), and survival compared to the FMFO reference diet. Notably, protein digestibility was highest in the 100% fish-free diets, suggesting efficient nutrient absorption and a potential synergistic effect between the two microalgal ingredients (Table S6). The apparent protein digestibility and amino acid profiles observed in this study are consistent with previous findings on microalgal feeds used to replace FM or FMFO in rainbow trout [19,27] and Atlantic salmon [25,28,49]. These results underscore the ability of a well-formulated microalgal diet to meet the essential nutritional requirements of rainbow trout, supporting the viability of fully fish-free aquafeeds without compromising physiological performance.

It is also noteworthy that apparent protein digestibility can be strongly influenced by fecal collection methodology, especially for microalgal-based diets, which tend to produce less cohesive feces with higher proportions of water-soluble nitrogen. In this study, fecal samples were collected using a modified settlement column approach based on the original Guelph system [39], with a radial flow settler installed between the culture tank outflow and sump tank inflow to facilitate rapid recovery of intact fecal matter [38]. Samples were immediately processed, the supernatant carefully removed, and feces frozen at −20 °C to minimize nutrient leaching, consistent with previously validated protocols for rainbow trout [27,38]. While some degree of overestimation of digestibility cannot be entirely excluded, the method is widely recognized as reliable for determining ADCs and avoids artifacts associated with more invasive collection techniques, such as manual stripping, which can introduce endogenous contamination, potentially underestimating digestibility [50,51]. High ADC values observed for the NSW100 and NSO100 diets may therefore reflect both the efficacy of the radial settler method and the characteristics of the feed, including extrusion processing, which disrupts cell walls and reduces anti-nutritional factors, enhancing nutrient availability [25,28,52]. Previous studies have also reported near-complete digestibility (~99%) of macronutrients, amino acids, and fatty acids in alternative ingredients such as microalgae and vital wheat gluten [53,54], supporting the plausibility of the high ADCs observed in this work. These findings highlight the importance of careful fecal recovery and feed processing when comparing digestibility among novel microalgal and conventional fishmeal-based diets.

A key factor likely contributing to the improved outcomes in this study is the inclusion of taurine in all treatment diets. Taurine, a known feeding stimulant, has been shown to enhance palatability and nutrient absorption in fish-free diets [55,56,57]. In our recent work, taurine supplementation significantly improved performance in rainbow trout fed fishmeal-free microalgal diets [19], and it likely played a similar role here by supporting consistent feed intake and utilization in the absence of FM and FO.

### 4.2. Replacement of Fish Oil with Schizochytrium sp.

This study also compared *Schizochytrium* sp. oil (NSO100) with its whole-cell counterpart (NSW100) as a DHA- and antioxidant-rich replacement for FO. Importantly, the comparison between NSW100 and NSO100 represents the only controlled single-factor comparison in this study, as both diets contained identical basal formulations and differed only in whether *Schizochytrium* was supplied as whole-cell biomass or extracted oil. Therefore, differences observed between these treatments can be attributed primarily to the physical form of the *Schizochytrium* ingredient and its associated nutritional or bioactive properties. In contrast, comparisons involving the FMFO and NSW75 diets should be interpreted as evaluations of practical dietary replacement strategies because they involve concurrent changes in multiple dietary components. This distinction should be considered when interpreting the biological responses among treatments. While NSO100 had the highest DHA content (6.11% of TFA) and high protein digestibility, fish in this group had lower growth compared to the FMFO diet. This was unexpected, as previous studies have found that DHA-rich diets often enhance growth performance in salmonids [46,48]. A potential explanation for the reduced growth in fish fed the NSO100 diet is its slightly lower EPA content (0.24% of TFA), which may contribute to imbalanced fatty acid profiles and reduced growth in trout and other carnivorous fish. This underscores the importance of maintaining an optimal DHA:EPA ratio, rather than maximizing DHA alone, for achieving favorable growth outcomes. The NSW100 diet contained a somewhat higher EPA level (0.34% of TFA), which may have played a minor role in the improved final weight and weight gain observed; however, the difference was not significant. In our diet formulation, lipids were primarily supplied by whole-cell biomass and oil from *Schizochytrium* sp. combined with canola oil to replace FO. Consequently, the experimental diets differed in fatty acid composition from the reference diet, particularly due to lower EPA + DHA levels; however, all diets were formulated to meet or exceed the essential fatty acid requirements of rainbow trout as established by [36]. This formulation offers a practical strategy to reduce reliance on finite marine resources while maintaining nutritional adequacy. Canola oil supplied energy and α-linolenic acid, complementing the DHA-rich *Schizochytrium* ingredients.

Despite differences in dietary fatty acid composition, whole-body fatty acid profiles remained remarkably stable across treatments. No significant differences were observed in DHA (22:6ω3), total ω3 PUFA, total PUFA, total ω3 LC-PUFA, or total ω6 LC-PUFA, with the exception of the ω3/ω6 ratio (Table 6). These results suggest that the experimental diets successfully maintained tissue fatty acid composition despite substantial reductions in marine-derived FO. The relatively stable tissue fatty acid profiles may partly reflect the capacity of freshwater species such as rainbow trout to elongate and desaturate α-linolenic acid into longer-chain ω3 fatty acids, including EPA and DHA [58], although this metabolic pathway cannot completely compensate for reduced dietary DHA supply [59]. While EPA and DHA metabolism was not directly investigated in this study, future research should investigate how microalgal ingredients can impact this pathway. This will be an important step in determining the most efficient and sustainable combination of microalgae to replace FO. Future research should optimize fatty acid profiles and clarify their digestion, metabolism, and tissue deposition in fish-free aquafeeds. Our findings demonstrate that microalgal ingredients can replace fish-derived feed components without compromising fish performance.

### 4.3. Antioxidant Compounds in Microalgal Ingredients and Diets

In addition to providing critical fatty acids, *Schizochytrium* sp. has emerged as a promising source of natural antioxidants due to its high content of phenolic compounds, antioxidant peptides, carotenoids (e.g., astaxanthin), and tocopherols—all of which have been shown to retain activity following processing and digestion [23,32,37,60]. Marine microalgae can provide functional benefits in aquafeeds beyond their protein and lipid contributions because of their diverse bioactive compounds, including carotenoids, xanthophylls, tocopherols, and phenolics. These compounds may enhance dietary oxidative stability and support antioxidant defenses in fish. Consistent with this potential, Karataş et al. (2025) reported that dietary *Chlorella* sp. and *Schizochytrium* sp. extracts supported growth and nutrient utilization while upregulating genes associated with antioxidant and immune functions in rainbow trout [30]. Similarly, Qiao et al. (2019) found that replacing fishmeal with 5% *Nannochloropsis* sp. meal significantly enhanced antioxidant capacity in juvenile turbot [31]. The presence of these antioxidant compounds may provide an additional functional advantage of replacing conventional fishmeal and fish oil with microalgal ingredients, particularly in PUFA-rich diets.

These compounds are increasingly linked to improved oxidative stability and enhanced immune function in aquafeeds [23,32,61]. This study analyzed the antioxidant content of key ingredients and the diets but did not include any analysis of antioxidant capacity or activity in rainbow trout. All diets in this experiment had similar levels of the antioxidants xanthophyll and vitamin E, but the NSO100 diet had less carotene than all other diets (Table 2). This is likely due to the numerically lower levels of carotene measured in the *Schizochytrium* sp. oil over the whole-cell (Table 3). It is also important to note that the *Nannochloropsis* by-product had greater carotene and vitamin E content than the fish meal used in this study. This antioxidant content should be investigated further as a benefit of microalgal ingredients in aquafeeds. Although antioxidant capacity in fish fillet was not directly evaluated in this study, our findings underscore the potential value of whole-cell *Schizochytrium* sp. in aquafeed formulations. This warrants further investigation, particularly as oxidative stability becomes a critical factor in the development of high-PUFA aquafeeds. Beyond its well-known role in salmonid pigmentation, compounds like astaxanthin from *Schizochytrium* sp. offer broader health and welfare benefits, affirming the microalga’s relevance as a multifunctional, sustainable feed ingredient in modern aquaculture [62].

Although algal oil lacks the indigestible cell wall that can hinder nutrient utilization [63], the NSO100 treatment did not outperform the whole-cell *Schizochytrium* sp. diets. This may be due to the low levels of carotene observed in the NSO100 treatment (Table 2). Our results found that the *Schizochytrium* sp. whole-cell had higher levels of carotene than *Schizochytrium* sp. oil (Table 3). This result suggests that bioactive compounds inherent in the intact biomass—such as tocopherols, carotenoids, structural lipids, and other functional molecules—may provide additional physiological benefits beyond those supplied by purified oil alone. The broader antioxidant profile associated with whole-cell microalgae may enhance cellular protection and improve oxidative stability [32,61,64]. The antioxidant activity and potential benefits were not directly measured in rainbow trout in this study. Future research should emphasize the impacts of microalgal ingredients on the antioxidant activity and physiology of rainbow trout and other key farmed fish.

### 4.4. Whole-Body Fatty Acid, Proximate, Amino Acid, and Nutrient Composition

In addition to evaluating growth performance, this study aimed to identify a fish-free feed alternative that maintains the nutritional quality of the fish. Previous research on plant-based aquafeeds has reported reduced deposition of essential fatty acids in fish tissues [16,17,20]. In the present study, the fatty acid profiles of whole-body samples closely reflected the fatty acid composition of the respective dietary treatments. Specifically, the microalgal-based diets exhibited numerically lower saturated fatty acid (SFA) and higher monounsaturated fatty acid (MUFA) levels compared to the FMFO diet (Table S1), with corresponding patterns observed in fish tissue deposition (Table 6). Given that sources low in saturated fatty acids (SFA) and high in monounsaturated fatty acids (MUFA) are considered beneficial for human health—particularly cardiovascular health [65,66]—the fatty acid profile observed in fish fed microalgal diets may represent a favorable nutritional outcome. Given the consistent pattern of SFA and MUFA levels between the experimental diets and whole-body samples—specifically, lower SFA and higher MUFA contents in both the test diets and the fish fed these diets compared with the reference group (Table S1 and Table 5)—the observed differences in tissue composition likely reflect the fatty acid profiles of the respective feeds.

With respect to polyunsaturated fatty acids (PUFA), this study found comparable levels of n-3 LC-PUFA (omega-3), n-6 LC-PUFA (omega-6), and docosahexaenoic acid (DHA) in the whole-body samples of fish fed fish-free, microalgal-based diets relative to those fed the FMFO-based reference diet. However, eicosapentaenoic acid (EPA) levels were significantly lower in the microalgal treatment groups. These whole-body fatty acid profiles closely mirrored the fatty acid composition of the respective diets (Table S1). The lower EPA levels in the microalgal diets were likely due to the use of *Nannochloropsis* by-product, from which a substantial portion of EPA had been extracted for human nutraceutical applications prior to feed formulation. Adequate dietary levels of n-3 LC-PUFA and n-6 LC-PUFA are critical to fish growth and health [67]. Despite the higher inclusion levels of n-3 long-chain polyunsaturated fatty acids (LC-PUFA) in the FMFO diet, no significant differences were observed in whole-body n-3 LC-PUFA content between treatment groups. This suggests either a higher digestibility and retention of n-3 LC-PUFA in the microalgal diets or a lower bioavailability of these fatty acids in the FMFO aquafeed. The findings on DHA and EPA content in this study align with previous research on trout fed microalgal replacement diets, where increasing levels of microalgae led to a reduction in EPA content while DHA levels remained unaffected [68,69].

The role of eicosapentaenoic acid (EPA) in fish growth and health is complex and species-specific. In Atlantic salmon, for instance, diets rich in docosahexaenoic acid (DHA) but low in EPA have been shown to impair growth when EPA levels fall below 1.3% of total fatty acids (TFA) [46]. In contrast, recent studies on rainbow trout have reported that diets containing EPA levels as low as 0.2–0.3% of TFA—similar to those in the microalgal-based diets used in this study—did not negatively impact growth performance, feed intake, survival, pigmentation, welfare indicators, histology, or fillet quality [48]. Despite these findings, the reduced growth observed in the NSO100 group compared to the FMFO reference group may suggest a growth-limiting role of EPA, particularly as NSO100 had the lowest dietary and whole-body EPA levels of all treatments and was one order of magnitude lower than in NSW75 and reference diets (Table S1). Notably, our data showed no evidence of retro-conversion of DHA to EPA, which has been previously reported as limited in salmonids but may increase under essential fatty acid (EFA) deficiency [70]. Additionally, high dietary DHA levels are known to inhibit the conversion of α-linolenic acid (ALA) to EPA and reduce EPA retention in fish tissues [71,72], which could partly explain the observed EPA shortfall in the NSO100 group. Our results did not indicate an overall EFA deficiency in the microalgal diets but highlight the need to define optimal EPA levels in trout nutrition. Future studies should optimize EPA relative to DHA in microalgal fish-free diets.

We found no difference in essential amino acids, proximate composition (except for protein), or trace elements in rainbow trout between dietary treatment groups. While plant-based diets have often been associated with deficiencies in essential amino acids [16,17], microalgae offer a more balanced amino acid profile, meeting the nutritional requirements of carnivorous fish species. Furthermore, unlike FMFO diets, which are prone to higher levels of toxic trace elements such as arsenic and mercury due to biomagnification through the marine food web [18,73,74,75], microalgal diets present a cleaner and potentially safer alternative. The *N. oculata* by-product used in this study had low levels of toxic trace elements (Table S4). A more comprehensive review is needed to confirm this trend across the microalgae production industry. The absence of elevated contaminants, combined with adequate nutrient composition, highlights the potential of microalgae as a sustainable and nutritionally sound feed ingredient for aquaculture.

### 4.5. Economic Implications and Economic Conversion Ratio

Fish-free diets had the lowest formulated feed cost, with NSW100 also yielding the lowest ECR, showing that fish-free feed using combinations of microalgal biomass could be a cost-competitive, large-scale alternative to FMFO for trout. Historically, microalgae replacement diets have not been a cost-competitive replacement for FMFO, in large part due to the high cost of microalgal oil. For example, in this study, costs ranged from 1.68 USD per kg for FO, 2.38 USD per kg for *Schizochytrium* sp. whole cell, and 4.41 USD per kg for *Schizochytrium* sp. oil. Nevertheless, despite the higher cost of microalgal oil, the overall formulated feed cost for microalgae-based diets was numerically lower, primarily due to the relatively high prices of FM (1.54 USD/kg) and FO (1.68 USD/kg) compared to the significantly lower costs of *N. oculata* by-product (0.54 USD/kg) and canola oil (0.88 USD/kg) (Table S5), and the higher quantities of FO required in reference diet compared to the DHA-rich microalgae diets (Table 1). As microalgae are increasingly sourced as underutilized by-products from adjacent industries such as biofuels and nutraceuticals, their production costs are expected to decline [27,76,77,78]. The economic feasibility of microalgal feeds will improve with wider industrial integration and scale-up. Cost projections aside, microalgal diets composed of *N. oculata* and *Schizochytrium* sp. have already been shown to outcompete FMFO, corroborating the results in this study [18].

Compared to other replacement diets, the estimated cost of the microalgal feed used in this study is more competitive than insect meal—estimated to be 50% more expensive than FM—yet remains less competitive than plant-based diets, which have an estimated formulated feed cost of 0.64 USD/kg compared to 0.86 USD/kg for the most cost-effective microalgal diet, NSO100, in this study [18,35,79]. In our recent study on FM replacement using *N. oculata* feed, the fully FM-replacing diet exhibited the lowest formulated feed cost ($0.88/kg feed) and economic conversion ratio (ECR) of $0.86 per kg of rainbow trout produced [19]. As production scales up, lower microalgal biomass costs could make microalgal aquafeeds more economically competitive. However, conventional plant-based feed costs often exclude externalities such as eutrophication, greenhouse gas emissions, and land use, potentially underestimating their true environmental costs [43,80,81]. Furthermore, the cultivation of marine photosynthetic microalgae like *N. oculata* can utilize waste streams from other industries, positioning it as a more sustainable alternative to plant-based aquafeeds [80,82,83,84,85].

While microalgae-based feeds currently remain more expensive than currently dominant ingredients such as FMFO or plant-based proteins [11], this landscape is shifting. The price of fishmeal has surged by over two hundred percent in the past two decades [86], narrowing the cost gap. Meanwhile, advances in cultivation technologies are driving down the production costs of microalgae, improving their economic feasibility [87]. Due to this rapidly changing environment, it is important to note that the CAST database used in this study was based on the period studied, rather than current market prices. As production methods continue to become more efficient, microalgae-based aquafeeds are expected to become increasingly cost-competitive [18]. One notable example is the widespread industry adoption of *Schizochytrium* sp., a heterotrophic, DHA-rich, and antioxidant-rich microalga, as a fish oil alternative in salmon feeds. This transition reflects both technological innovation and growing consumer and market demand for sustainable sources of omega-3 fatty acids. Moreover, the cost of feed ingredients is closely tied to production scale; as adoption increases, economies of scale are likely to further reduce costs.

Several major players in the aquafeed and agribusiness sectors—including Corbion, BioMar, Archer Daniels Midland (ADM), and Veramaris—have significantly expanded their efforts to develop and commercialize fish oil substitutes based on *Schizochytrium* sp. [21,38,88]. Unlike photosynthetic microalgae, *Schizochytrium* can be grown heterotrophically in industrial bioreactors without the need for sunlight or carbon dioxide, thereby reducing production costs [88,89] and reduces certain though not all life-cycle environmental impacts [81]. Its cultivation is performed in controlled environments, which limits ecological disruption and enhances sustainability [27,90]. An open environmental life-cycle analysis showed that *Schizochytrium*-based omega-3 production offers two environmental benefits: elimination of dependency on wild-caught forage fish and lower greenhouse gas emissions [81]. These advantages position *Schizochytrium* as a cornerstone of future aquafeed formulations and align closely with the broader goals of ecological stewardship and responsible aquaculture.

The findings of this study underscore the cost-competitiveness and practical viability of fish-free microalgal diets in aquaculture. However, one limitation of this study in estimating the ECR is that the CAST does not account for the costs of certain minor ingredients, such as taurine and lecithin, used in this experiment. Additionally, expenses related to feed manufacturing, labor, transportation, and equipment were excluded from the analysis, and projected ingredient prices may not fully reflect actual market conditions. While the ECR values derived in this study are encouraging, achieving cost competitiveness of microalgal ingredients relative to conventional feed components remains a key challenge. Ultimately, large-scale production and cost reductions through technological innovation and industrial scale-up will be critical to realizing the commercial viability of microalgae-based aquafeeds.

Expanding the portfolio of microalgal raw materials through innovative approaches presents significant opportunities to improve formulation flexibility—prioritizing optimal nutrition, sustainability, availability, and affordability [35]. Realizing the full potential of microalgae as a sustainable feed ingredient depends on scaling up production processes to balance environmental responsibility with economic viability. The inherent biological advantages of microalgae, combined with mounting evidence supporting their effectiveness as a replacement protein and lipid source across diverse aquaculture species, position them as a promising alternative in the sector. Nonetheless, large-scale, continuous production of high-quality microalgal biomass remains challenged by technical, biological, and economic barriers—particularly in optimizing downstream processing and ensuring consistent biomass quality [91,92].

### 4.6. Future Directions

This study demonstrates that fish-free diets formulated with *Nannochloropsis oculata* by-product and DHA-rich *Schizochytrium* sp. whole cells can fully replace traditional FMFO in juvenile rainbow trout aquafeeds—with similar growth performance, protein digestibility (which increased), feed conversion ratio, and nutrient deposition. Importantly, our cost analysis supports the potential for these microalgae by-product-based diets to be cost-competitive with conventional FMFO feeds, possibly before projected cost reductions associated with industry-scale production of algal ingredients. This challenges the long-standing assumption that microalgal feeds are prohibitively expensive and positions them as a practical solution for commercial adoption.

Together, these findings reinforce the transformative potential of microalgae as a sustainable, nutritionally adequate, and economically viable alternative to FMFO in aquaculture. Future efforts should focus on optimizing inclusion levels, improving processing efficiency, and expanding the portfolio of algal by-products available for feed formulation. Only through real-world commercial on-farm validation can microalgae-based diets’ full economic, environmental, and operational benefits be realized at scale—supporting not only aquaculture productivity but also the global imperative to reduce dependence on wild-caught forage fish and promote a more resilient, ocean-friendly food system.

## Figures and Tables

**Table 1:** Formulation (g/100 g diet) and essential amino acids (% in the weight of diet) of four experimental diets for juvenile rainbow trout.

Ingredient (%)	Diet			
	Reference ^a^	NSW75 ^b^	NSW100 ^c^	NSO100 ^d^
Fish meal ^e^	7.5	1.875	0	0
Fish oil	14	3.5	0	0
*N. oculata* by-product	0	6	8	8
*Schizochytrium* (whole cells)	0	5	5	0
*Schizochytrium* (oil)	0	0	0	2.8
Canola oil	0	9	12	12
Feather meal	15	15	15	15
Blood meal	7	7	7	7
Corn gluten meal	20	20	20	20
Soy protein concentrate	20	20	20	20
Wheat gluten	5	5	5	5
CaHPO_4_	1	1	1	1
Vitamin-mineral premix ^f^	0.6	0.6	0.6	0.6
Lysine	1	1	1	1
Methionine	0.2	0.2	0.2	0.2
Choline chloride	0.5	0.5	0.5	0.5
Wheat flour	4.5	0.58	1	3.2
Ascorbic acid	0.2	0.2	0.2	0.2
Astaxanthin	0.05	0.05	0.05	0.05
Soy lecithin	3	3	3	3
Taurine	0.5	0.5	0.5	0.5

^a^ Reference: no replacement of fish meal (FM) and fish oil (FO). ^b^ Replacement of 75% FMFO with *N. oculata* and *Schizochytrium* whole cells. ^c^ Replacement of 100% of FMFO with *N. oculata* and *Schizochytrium* whole cells. ^d^ Replacement of 100% of FMFO with *N. oculata* and *Schizochytrium* oil. ^e^ Omega Protein, Inc., Houston, Texas, as manufacturer specification, the guaranteed gross composition analysis: crude protein, 60%; crude fat, 6%; fiber, 2%. ^f^ Mineral premix (mg kg^−1^ dry diet unless otherwise stated): ferrous sulfate, 0.13; NaCl, 6.15; copper sulfate, 0.06; manganese sulfate, 0.18; potassium iodide, 0.02; zinc sulfate, 0.3; carrier (wheat middling or starch).

**Table 2:** Proximate composition, essential amino acids, and antioxidant content of diets.

	Diet ^a^			
	Reference ^b^	NSW75 ^c^	NSW100 ^d^	NSO100 ^e^
Proximate composition (%)				
Moisture	17.37	16.03	17.02	18.46
Crude protein	47.32	47.58	46.82	46.4
Lipid	15.28	16.2	15.39	15.52
Ash	4.92	4.84	4.6	4.25
Fiber	1.14	1.65	1.66	1.63
Carbohydrates	15.11	15.36	16.18	15.36
Energy (Kcal/kg)	3443.33	3521.67	3454.67	3424.33
Essential amino acids (%)				
Arginine	2.69	2.53	2.65	2.37
Lysine	2.73	2.67	2.71	2.78
Isoleucine	1.87	1.68	1.84	1.5
Leucine	4.61	4.53	4.6	4.4
Histidine	1.16	1.05	1.06	0.98
Methionine	0.77	0.85	0.83	0.83
Phenylalanine	2.53	2.53	2.51	2.46
Threonine	1.94	1.97	1.92	1.79
Tryptophan	0.42	0.38	0.41	0.35
Valine	2.75	2.53	2.64	2.00
Antioxidant content ^f^				
Carotene (mg/kg)	1.3	1.7	1.1	0.4
Xanthophyll (mg/kg)	3.9	3.1	2.6	2.6
Vitamin E (IU/kg)	230	224	193	207

^a^ Values are means of three replicate groups (*n* = 3). ^b^ Reference: no replacement of fish meal (FM) and fish oil (FO). ^c^ Replacement of 75% FMFO with *N. oculata* and *Schizochytrium* whole cells. ^d^ Replacement of 100% of FMFO with *N. oculata* and *Schizochytrium* whole cells. ^e^ Replacement of 100% of FMFO with *N. oculata*, *Schizochytrium* oil, and canola oil. ^f^ Values are means of two replicate groups (*n* = 2).

**Table 3:** Proximate compositions, amino acid profiles, and antioxidant content of *N. oculata* defatted co-product, *Schizochytrium* sp., Fish Meal, and Fish oil.

	Ingredients				
	*N. oculate* ^a^	Schizo WC ^b^	Schizo Oil (Fat)	Fish Meal ^c^	Fish Oil
Proximate composition (g/100 g as is)					
Crude protein	49.7	11.9	N/A	63.81	N/A
Lipid	4.8	54.1	101.6	11.64	100.5
Ash	8.6	8.7	N/A	19.29	N/A
Fiber	0.37	2.4	N/A	0.44	N/A
Energy, kJ g^−1^	11.23	17.7	N/A	13.55	N/A
Essential amino acids (g/100 g in the weight of ingredient as is)					
Arginine	2.6	0.08	N/A	4.6	N/A
Lysine	2.7	0.05	N/A	5.0	N/A
Isoleucine	2.1	0.04	N/A	2.1	N/A
Leucine	4.2	0.07	N/A	3.8	N/A
Histidine	0.9	0.03	N/A	1.4	N/A
Methionine	1.0	0.12	N/A	1.3	N/A
Phenylalanine	2.4	0.04	N/A	2.4	N/A
Threonine	2.4	0.04	N/A	2.7	N/A
Tryptophan	0.5	0.02	N/A	0.2	N/A
Valine	2.9	0.06	N/A	2.7	N/A
Fatty acids fractions (% TFA)					
Total SFA	28.53	9.2	28.94	27.44	6.6
Total MUFA	34.34	1.7	0.65	54.14	23.9
Total PUFA	37.13	63.7	70.41	19.65	40.6
20:5n-3 EPA	27.94	0.8	0.43	1.95	14.9
22:6n-3 DHA	*ND*	43.2	57.90	3.03	13
Total n-3 PUFA	28.03	45.9	59.40	1.95	36.3
Total n-6 PUFA	8.71	17.8	11.01	3.03	4.3
Antioxidant content					
Carotene (mg/kg)	9.0	23.2	0.2	1.1	1.1
Xanthophyll (mg/kg)	N/A	5.2	0.3	N/A	N/A
Vitamin E (IU/kg)	31	<25	1700	<25	N/A

^a^ Proximate, amino acid, and fatty acid data previously reported in [18] ^b^ Proximate, amino acid, and fatty acid data previously reported in [37] ^c^ Proximate and amino acid data previously reported in [19].

**Table 4:** Growth indices (mean ± standard error, *n* = 4) of rainbow trout fed experimental diets for 84 days.

Growth Indices	Diet ^a^				ANOVA	
	Reference ^b^	NSW75 ^c^	NSW100 ^d^	NSO100 ^e^	*F* Value	*p* Value
Initial Wt. (g)	0.84 ± 0.03	0.83 ± 0.03	0.91 ± 0.06	0.84 ± 0.03	0.945	0.45
Final Wt. (g)	35.0 ± 0.26 ^f^	33.0 ± 0.77 ^fg^	34.3 ± 0.48 ^fg^	32.9 ± 0.26 ^g^	4.277	0.029
Wt. gain (g)	34.2 ± 0.24 ^f^	32.2 ± 0.77 ^fg^	33.4 ± 0.51 ^fg^	32.0 ± 0.25 ^g^	4.227	0.03
Wt. gain (%)	4084.7 ± 149.21	3886.7 ± 144.86	3701.8 ± 255.86	3840.5 ± 150.61	0.764	0.536
Feed intake (g)	30.2 ± 0.9	29.7 ± 0.3	29.8 ± 0.5	31.4 ± 1.0	1.130	0.376
FCR	0.88 ± 0.03	0.93 ± 0.03	0.89 ± 0.02	0.98 ± 0.03	2.686	0.094
SGR	4.4 ± 0.04	4.4 ± 0.04	4.3 ± 0.08	4.4 ± 0.05	0.778	0.529
PER	2.4 ± 0.08	2.3 ± 0.07	2.4 ± 0.06	2.2 ± 0.06	2.719	0.091
Survival (%)	100	100	100	100	n/a	n/a

^a^ Values are means ± standard errors of four replicate groups (*n* = 4). ^b^ Reference: no replacement of fish meal (FM) and fish oil (FO). ^c^ Replacement of 75% of FMFO with *N. oculata* and *Schizochytrium* whole cells. ^d^ Replacement of 100% of FMFO with *N. oculata* and *Schizochytrium* whole cells. ^e^ Replacement of 100% of FMFO with *N. oculata*, *Schizochytrium* oil, and canola oil. ^f,g^ Mean values not sharing a superscript letter in the same row differ significantly (*p* < 0.05) by Tukey’s HSD test.

**Table 5:** Whole-body proximate composition (dry weight basis) of rainbow trout after 84 days on the experimental diets.

Proximate Composition (%)	Whole-body ^a^				ANOVA	
	Reference ^b^	NSW75 ^c^	NSW100 ^d^	NSO100 ^e^	*F* Value	*p* Value
Moisture	76.89 ± 1.18	74.91 ± 2.13	78.89 ± 0.5	82.68 ± 1.73	3.248	0.060
Protein	50.06 ± 0.99 ^fg^	48.36 ± 0.72 ^f^	53.06 ± 0.62 ^g^	52.77 ± 0.66 ^g^	8.791	0.002
Fat	34.6 ± 0.58	36.87 ± 0.96	35.02 ± 0.7	34.29 ± 0.38	2.804	0.085
Fiber	1.42 ± 0.28	1.68 ± 0.64	1.34 ± 0.28	2.01 ± 0.54	0.421	0.741
Ash	5.02 ± 0.18	5.22 ± 0.25	4.9 ± 0.17	4.78 ± 0.2	0.835	0.500

^a^ Values are means ± standard errors of four replicate groups (*n* = 4). ^b^ Reference: no replacement of fish meal (FM) and fish oil (FO). ^c^ Replacement of 75% FMFO with *N. oculata* and *Schizochytrium* whole cells. ^c^ Replacement of 100% of FMFO with *N. oculata* and *Schizochytrium* whole cells. ^e^ Replacement of 100% of FMFO with *N. oculata*, *Schizochytrium* oil, and canola oil. ^f,g^ Mean values not sharing a superscript letter in the same row differ significantly (*p* < 0.05) by Tukey’s HSD test.

**Table 6:** Fatty acid content of whole-body rainbow trout after 84 days on experimental diets.

Fatty Acid (% TFA) ^a^	Whole-Body				ANOVA	
	Reference ^b^	NSW75 ^c^	NSW100 ^d^	NSO100 ^e^	F Value	*p* Value
14:0	7.18 ± 0.37 ^l^	2.59 ± 0.12 ^m^	1.68 ± 0.1 ^n^	0.93 ± 0.08 ^n^	191.619	<0.001
15:0	0.68 ± 0.04 ^l^	0.25 ± 0.02 ^m^	0.19 ± 0.01 ^m^	0.16 ± 0.01 ^m^	112.803	<0.001
16:0	30.96 ± 1.18 ^l^	21.46 ± 0.39 ^m^	19.18 ± 0.46 ^m^	18.57 ± 0.64 ^m^	61.113	<0.001
17:0	0.74 ± 0.04 ^l^	0.28 ± 0.01 ^m^	0.21 ± 0.01 ^mn^	0.19 ± 0.02 ^n^	147.417	<0.001
18:0	7.36 ± 0.34 ^l^	5.81 ± 0.22 ^m^	5.37 ± 0.11 ^m^	4.91 ± 0.19 ^m^	21.31	<0.001
20:0	0.45 ± 0.02 ^l^	0.52 ± 0.02 ^m^	0.55 ± 0.01 ^m^	0.52 ± 0.02 ^m^	6.609	0.007
22:0	0.28 ± 0.01	0.29 ± 0.01	0.3 ± 0.01	0.28 ± 0.01	0.841	0.497
24:0	0.21 ± 0.01	0.26 ± 0.02	0.24 ± 0.02	0.25 ± 0.03	1.169	0.362
Total Saturated Fatty Acids	47.98 ± 2 ^l^	31.51 ± 0.75 ^m^	27.72 ± 0.55 ^mn^	25.84 ± 0.88 ^n^	72.194	<0.001
16:1ω9	0.35 ± 0.01 ^l^	0.3 ± 0.01 ^m^	0.31 ± 0.01 ^m^	0.3 ± 0.01 ^m^	8.133	0.003
16:1ω7	10.54 ± 0.29 ^l^	3.25 ± 0.04 ^m^	1.82 ± 0.12 ^n^	1.51 ± 0.12 ^n^	624.081	<0.001
18:1ω9	26.25 ± 0.67 ^l^	50.68 ± 0.32 ^m^	55.67 ± 0.38 ^n^	55.33 ± 0.88 ^n^	536.128	<0.001
18:1ω7	4.54 ± 0.06 ^l^	3.54 ± 0.06 ^m^	3.33 ± 0.04 ^mn^	3.25 ± 0.03 ^n^	139.064	<0.001
20:1ω9	1.78 ± 0.06 ^l^	2.3 ± 0.08 ^mn^	2.51 ± 0.05 ^m^	2.15 ± 0.09 ^n^	18.471	<0.001
20:1ω7	0.39 ± 0.09	0.67 ± 0.09	0.66 ± 0.04	0.59 ± 0.1	2.399	0.119
22:1ω11	0.14 ± 0.05	0.2 ± 0.02	0.22 ± 0.08	0.24 ± 0.02	0.678	0.582
22:1ω9	0.5 ± 0.05 ^l^	0.68 ± 0.05 ^lm^	0.75 ± 0.03 ^m^	0.76 ± 0.05 ^m^	7.35	0.005
24:1ω9	0.46 ± 0.03	0.39 ± 0.04	0.37 ± 0.02	0.41 ± 0.03	1.307	0.317
Total Monounsaturated Fatty Acids ^g^	45.39 ± 0.37 ^l^	62.18 ± 0.42 ^m^	65.77 ± 0.39 ^n^	64.62 ± 1.05 ^mn^	231.291	<0.001
18:2ω6	4.19 ± 1.28	4.89 ± 0.72	5.13 ± 0.4	7.43 ± 1.47	1.76	0.208
18:3ω6	0.02 ± 0.02	0 ± 0	0 ± 0	0.06 ± 0.02	3.134	0.066
20:2ω6	0.22 ± 0.07	0.26 ± 0.03	0.32 ± 0.01	0.37 ± 0.06	1.673	0.225
20:3ω6	0.05 ± 0.03	0.01 ± 0.01	0.04 ± 0.02	0.08 ± 0.03	1.086	0.392
20:4ω6 Arachidonic Acid	0.11 ± 0.05	0.14 ± 0.02	0.13 ± 0.01	0.14 ± 0.01	0.256	0.855
22:4ω6	0 ± 0	0 ± 0	0 ± 0	0 ± 0	.	.
22:5ω6	0.02 ± 0.02	0.1 ± 0.04	0.11 ± 0.02	0.1 ± 0.02	2.41	0.118
Total ω6 PUFA ^f^	4.61 ± 1.45	5.41 ± 0.78	5.73 ± 0.46	8.18 ± 1.62	1.699	0.22
18:3ω3 Alpha-Linolenic Acid ^j^	0.23 ± 0.1	0.24 ± 0.05	0.22 ± 0.04	0.49 ± 0.14	2.1	0.154
18:4ω3	0.07 ± 0.04	0 ± 0	0 ± 0	0.01 ± 0.01	2.097	0.154
20:3ω3	0 ± 0	0.02 ± 0.02	0 ± 0	0 ± 0	1	0.426
20:4ω3	0.03 ± 0.03	0 ± 0	0 ± 0	0 ± 0	1	0.426
20:5ω3 Eicosapentaenoic Acid	0.38 ± 0.11 ^l^	0.07 ± 0.01 ^m^	0 ± 0 ^m^	0.02 ± 0.02 ^m^	8.767	0.002
22:5ω3	0.06 ± 0.06	0 ± 0	0 ± 0	0 ± 0	1	0.426
22:6ω3 Docosahexaenoic acid (DHA)	0.91 ± 0.2	0.58 ± 0.04	0.55 ± 0.02	0.79 ± 0.06	2.644	0.097
Total ω3 PUFA ^g^	1.67 ± 0.53	0.91 ± 0.02	0.78 ± 0.06	1.32 ± 0.23	1.952	0.175
Total PUFA ^h^	6.63 ± 2.07	6.32 ± 0.78	6.51 ± 0.51	9.54 ± 1.81	1.11	0.383
Total ω6 LC-PUFA ^i^	0.4 ± 0.16	0.51 ± 0.06	0.6 ± 0.06	0.68 ± 0.12	1.208	0.349
Total ω3 LC-PUFA ^j^	1.38 ± 0.4	0.67 ± 0.05	0.55 ± 0.02	0.82 ± 0.08	3.091	0.068
ω3/ω6 PUFA ^k^	0.37 ± 0.03 ^l^	0.18 ± 0.03 ^m^	0.14 ± 0 ^m^	0.17 ± 0.01 ^m^	23.598	<0.001

^a^ Total fatty acids (TFA) (%); Mean ± standard error for 4 replicates per diet (pooled whole tissues of 3 fish/replicate). ^b^ Reference: no replacement of fish meal (FM) and fish oil (FO). ^c^ Replacement of 75% FMFO with *N. oculata* and *Schizochytrium* whole cells. ^d^ Replacement of 100% of FMFO with *N. oculata* and *Schizochytrium* whole cells. ^e^ Replacement of 100% of FMFO with *N. oculata*, *Schizochytrium* oil, and canola oil. ^f^ Omega-6 (n-6) Polyunsaturated fatty acids (PUFAs) (sum of all fatty acids with ≥2 double bonds (18:2, 18:3, 20:2, 20:3, 20:4, 2:4, 22:5). ^g^ Omega-3 PUFAs (18:3, 18:4, 20:3, 20:4, 20:5, 22:5, 22:6). ^h^ Total PUFA is the sum of all fatty acids with two or more double bonds. ^i^ n-6 long-chain (LC) PUFA (20:2, 20:3, 20:4, 22:4, 22:5). ^j^ n-3 LCPUFA (20:3, 20:4, 20:5, 22:5, 22:6). ^k^ Ratio calculated for total n-3 PUFA: total n-6 PUFA (n-3/n-6). ^l,m,n^ Mean values across the row not sharing a common superscript were significantly different as determined by Tukey’s HSD test, *p* < 0.05.

**Table 7:** Proximate fillet composition (dry weight basis) of rainbow trout fed experimental diets for 84 days.

Proximate Composition (%)	Fillet ^a^				ANOVA	
	Reference ^b^	NSW75 ^c^	NSW100 ^d^	NSO100 ^e^	*F* Value	*p* Value
Moisture	81.06 ± 0.75	81.13 ± 1.76	83.74 ± 1.58	81.08 ± 0.78	1.088	0.391
Protein	69.9 ± 1.33	74.43 ± 1.01	75.85 ± 1.75	69.7 ± 3.01	2.650	0.096
Fat	15.6 ± 1.34	14.68 ± 1.54	14.3 ± 1.26	20.37 ± 3.02	2.118	0.151
Fiber	1.52 ± 0.8	0.45 ± 0.18	1.23 ± 0.55	0.58 ± 0.2	1.051	0.406
Ash	5.18 ± 0.12	5.18 ± 0.12	5.15 ± 0.18	4.74 ± 0.12	2.441	0.115

^a^ Values are means ± standard errors of four replicate tanks (*n* = 4) ^b^ Reference: no replacement of fish meal (FM) and fish oil (FO). ^c^ Replacement of 75% of FMFO with *N. oculata* and *Schizochytrium* whole cells. ^d^ Replacement of 100% of FMFO with *N. oculata* and *Schizochytrium* whole cells. ^e^ Replacement of 100% of FMFO with *N. oculata*, *Schizochytrium* oil, and canola oil.

**Table 8:** Essential amino acid content (dry weight basis) of fillets from rainbow trout after 84 days on the experimental diets.

Essential Amino Acids (%)	Fillet ^a^				ANOVA	
	Reference ^b^	NSW75 ^c^	NSW100 ^d^	NSO100 ^e^	*F* Value	*p* Value
Methionine	1.85 ± 0.06	2 ± 0.06	2 ± 0.08	1.94 ± 0.05	1.189	0.355
Lysine	5.87 ± 0.16	5.89 ± 0.35	6.01 ± 0.33	5.85 ± 0.54	0.037	0.990
Phenylalanine	2.62 ± 0.04	2.69 ± 0.14	2.7 ± 0.12	2.8 ± 0.11	0.474	0.706
Leucine	4.98 ± 0.09	5.06 ± 0.26	5.14 ± 0.24	5.02 ± 0.4	0.064	0.978
Isoleucine	2.57 ± 0.21	2.45 ± 0.38	2.24 ± 0.3	2.35 ± 0.5	0.159	0.922
Threonine	3.05 ± 0.07	3.11 ± 0.21	3.08 ± 0.19	3.06 ± 0.26	0.017	0.997
Valine	2.9 ± 0.22	2.89 ± 0.34	2.7 ± 0.27	2.99 ± 0.36	0.158	0.922
Histidine	2.02 ± 0.03	2.06 ± 0.09	2.13 ± 0.09	2.06 ± 0.09	0.305	0.821
Arginine	4.09 ± 0.1	4.21 ± 0.24	4.18 ± 0.2	3.97 ± 0.37	0.183	0.906

^a^ Values are means ± standard errors of four replicate tanks (*n* = 4) ^b^ Reference: no replacement of fish meal (FM) and fish oil (FO). ^c^ Replacement of 75% FMFO with *N. oculata* and *Schizochytrium* whole cells. ^d^ Replacement of 100% of FMFO with *N. oculata* and *Schizochytrium* whole cells. ^e^ Replacement of 100% of FMFO with *N. oculata*, *Schizochytrium* oil, and canola oil.

**Table 9:** Macromineral and trace element content (dry weight basis) of whole body from rainbow trout after 84 days on the experimental diets.

	Whole-body ^a^				ANOVA	
	Reference ^b^	NSW75 ^c^	NSW100 ^d^	NSO100 ^e^	*F* Value	*p* Value
Macro minerals (%)						
Phosphorus	1.00 ± 0.06	1.08 ± 0.03	1.11 ± 0.06	0.99 ± 0.05	1.467	0.273
Calcium	0.67 ± 0.09	0.85 ± 0.07	0.78 ± 0.1	0.59 ± 0.09	1.784	0.204
Magnesium	0.08 ± 0.003	0.08 ± 0.001	0.09 ± 0.004	0.08 ± 0.001	1.974	0.172
Potassium	1.04 ± 0.04	1.05 ± 0.03	1.13 ± 0.02	1.1 ± 0.01	2.523	0.107
Sulfur	0.63 ± 0.02	0.66 ± 0.02	0.69 ± 0.02	0.69 ± 0.01	2.925	0.077
Trace elements (mg kg^−1^)						
Copper ^f^	ND	ND	ND	ND	.	.
Iron	33.6 ± 2.6	47.4 ± 8.6	41.9 ± 4.1	42.7 ± 1.2	1.342	0.307
Manganese ^f^	3.06 ± 0.19	3.46 ± 0.64	3.34 ± 0.31	2.89 ± 0.14	0.478	0.704
Selenium ^f^	ND	ND	ND	ND	.	.
Zinc	34.39 ± 1.46	40.01 ± 0.73	45.58 ± 5.05	71.55 ± 29.3	1.221	0.345
Arsenic ^f^	ND	ND	ND	ND	.	.
Boron ^f^	ND	ND	ND	ND	.	.
Aluminum	0.33 ± 0.67	10.41 ± 5.22	6.22 ± 2.31	9.15 ± 3.77	1.707	0.218
Mercury ^f^	ND	ND	ND	ND	-	-
Lead ^f^	ND	ND	ND	ND	.	.
Molybdenum ^f^	ND	ND	ND	ND	.	.

^a^ Values are means ± standard errors of four replicate tanks (*n* = 4); each replicate involves pooled whole tissues of 3 fish. ^b^ Reference: no replacement of fish meal (FM) and fish oil (FO). ^c^ Replacement of 75% of FMFO with *N. oculata* and *Schizochytrium* whole cells. ^d^ Replacement of 100% of FMFO with *N. oculata* and *Schizochytrium* whole cells. ^e^ Replacement of 100% of FMFO with *N. oculata*, *Schizochytrium* oil, and canola oil. ^f^ Not detectable (ND) (<0.00 mg/kg).

**Table 10:** Formulated feed cost, feed conversion ratio, and economic conversion ratio of rainbow trout production.

Cost Metrics	Scenario ^a^				ANOVA	
	Reference ^c^	NSW75 ^d^	NSW100 ^e^	NSO100 ^f^	F Value	*p* Value
Formulated Feed cost ^b^	0.97	0.92	0.87	0.88	N/A	N/A
FCR	0.88 ± 0.03	0.93 ± 0.03	0.89 ± 0.02	0.98 ± 0.03	2.776	0.087
ECR	0.86 ± 0.03	0.85 ± 0.03	0.77 ± 0.02	0.86 ± 0.02	2.544	0.105

^a^ Mean ± standard error for 4 replicates per diet. ^b^ Formulated Feed cost (FFC) = cost of feed ($)/kg feed. ^c^ Reference: no replacement of fish meal (FM) and fish oil (FO). ^d^ Replacement of 75% FMFO with *N. oculata* and *Schizochytrium* whole cells. ^e^ Replacement of 100% of FMFO with *N. oculata* and *Schizochytrium* whole cells. ^f^ Replacement of 100% of FMFO with *N. oculata*, *Schizochytrium* oil, and canola oil.

## Data Availability

All data generated for this study are available at the USDA Ag Data Commons repository: https://doi.org/10.15482/USDA.ADC/32290524.

## Supplementary Materials

The following supporting information can be downloaded at: https://www.mdpi.com/article/10.3390/antiox15101343/s1, Table S1. Fatty acid profiles of dietary treatments, Table S2. Macrominerals and trace elements of dietary treatments. Table S3. Total fatty acid profile by percentage of *N. oculata* defatted biomass, *Schizochytrium* sp. whole cells, and *Schizochytrium* DHA oil. Table S4. Macromineral and trace element composition of *N. oculata* defatted biomass, *Schizochytrium* sp. whole cells, and *Schizochytrium* sp. oil. Table S5. Experimental ingredient market prices obtained from Cruz Aquafeed Sustainability Tool (CAST). Table S6. Digestibility of crude protein between dietary treatments. Table S7. Amounts of lipid and major omega-3 (n-3) and n-6 polyunsaturated fatty acids (PUFAs) of rainbow trout fed dietary treatments for 84 days.
