# Supplementary material for: Toward Sustainable Aquaculture: Microalgae Fully Replace Fishmeal and Fish Oil in Rainbow Trout Diets While Maintaining Growth, Nutritional Quality, and Cost Viability
Source: Antioxidants (Basel). 2026 Oct 16;15(10):1343. doi: 10.3390/antiox15101343 (PMC13701610; doi:10.3390/antiox15101343)
Supplement: Supplementary file 1 [file antioxidants-15-01343-s001.zip › Supplementary-Materials_Fish-free feed grwoth_nutrition_ECR__09.28.26.pdf]

Supplementary Materials

Table S1. Fatty acid profiles of dietary treatments.

	Diet TFA%[a]			
	Reference[b]	NSW75 [c]	NSW100 [d]	NSO100[e]
14:0	5.75 ± 0.15	1.9 ± 0.05	0.89 ± 0.01	0.42 ± 0.01
15:0	0.5 ± 0.01	0.16 ± 0	0.08 ± 0	0.06 ± 0
16:0	20.42 ± 0.08	12.88 ± 0.06	10.71 ± 0.03	11.35 ± 0.09
17:0	0.6 ± 0.01	0.2 ± 0	0.1 ± 0	0.1 ± 0
18:0	4.25 ± 0.05	2.96 ± 0.03	2.57 ± 0.03	2.66 ± 0.01
20:0	0.35 ± 0	0.49 ± 0.01	0.51 ± 0	0.53 ± 0.01
22:0	0.24 ± 0	0.29 ± 0.01	0.3 ± 0	0.31 ± 0.01
24:0	0.21 ± 0	0.26 ± 0	0.26 ± 0.02	0.24 ± 0
Total SFA[f]	32.39 ± 0.17	19.16 ± 0.07	15.43 ± 0.08	15.67 ± 0.07
16:1ω9	0.18 ± 0.01	0.06 ± 0	0.04 ± 0	0.02 ± 0.01
16:1ω7	9.66 ± 0.1	2.32 ± 0.02	0.64 ± 0.01	0.64 ± 0.01
18:1ω9	13.52 ± 0.13	38.17 ± 0.25	45.28 ± 0.07	44.48 ± 0.2
18:1ω7	3.03 ± 0.03	2.54 ± 0.02	2.46 ± 0	2.46 ± 0.01
20:1ω9	0.75 ± 0.01	0.75 ± 0.02	0.74 ± 0.01	0.74 ± 0.01
20:1ω7	0.13 ± 0	0 ± 0	0 ± 0	0 ± 0
22:1ω11	0.09 ± 0.01	0.08 ± 0.02	0.06 ± 0	0.05 ± 0
22:1ω9	0.21 ± 0	0.12 ± 0.02	0.09 ± 0.01	0.1 ± 0
24:1ω9	0.29 ± 0.02	0.42 ± 0.02	0.33 ± 0.02	0.13 ± 0.01
Total MUFA[g]	28.18 ± 0.07	44.6 ± 0.26	49.69 ± 0.07	48.66 ± 0.19
18:2ω6	15.49 ± 0.09	21.42 ± 0.07	23.91 ± 0.07	23.19 ± 0.13
18:3ω6	0.21 ± 0	0.06 ± 0	0 ± 0	0 ± 0
20:2ω6	0.23 ± 0	0.13 ± 0.02	0.07 ± 0.01	0.07 ± 0
20:3ω6	0.18 ± 0	0.09 ± 0	0.05 ± 0	0 ± 0
20:4ω6 ARA[h]	0.79 ± 0.01	0.4 ± 0	0.25 ± 0	0.17 ± 0.02
22:4ω6	0.13 ± 0	0.03 ± 0.02	0 ± 0	0 ± 0
22:5ω6	0.29 ± 0	1.47 ± 0.01	1.4 ± 0	1.18 ± 0.01
Total ω6 PUFA[i]	17.32 ± 0.09	23.6 ± 0.07	25.67 ± 0.06	24.62 ± 0.12
18:3ω3 ALA[j]	1.82 ± 0.02	3.93 ± 0.03	4.84 ± 0.05	4.62 ± 0.03
18:4ω3	1.37 ± 0.02	0.31 ± 0.01	0.02 ± 0.02	0 ± 0
20:3ω3	0.13 ± 0	0 ± 0	0 ± 0	0 ± 0
20:4ω3	0.74 ± 0.02	0.23 ± 0	0.07 ± 0	0.07 ± 0

20:5ω3 EPA[k]	7.65 ± 0.02	1.96 ± 0.02	0.34 ± 0	0.24 ± 0
22:5ω3	1.47 ± 0.02	0.36 ± 0	0 ± 0	0 ± 0
22:6ω3 DHA[l]	6 ± 0.04	5.23 ± 0.05	3.93 ± 0.01	6.11 ± 0.05
Total ω3 PUFA[m]	19.51 ± 0.07	12.11 ± 0.11	9.21 ± 0.08	11.05 ± 0.08
Total PUFA[n]	39.43 ± 0.14	36.24 ± 0.2	34.88 ± 0.13	35.67 ± 0.2
Total ω6 LC PUFA[o]	1.62 ± 0.01	2.12 ± 0.04	1.76 ± 0.01	1.42 ± 0.03
Total ω3 LC PUFA[p]	16.32 ± 0.08	7.86 ± 0.07	4.35 ± 0.01	6.43 ± 0.05
ω3 / ω6 PUFA[q]	1.13 ± 0	0.51 ± 0	0.36 ± 0	0.45 ± 0

[a] Mean ± standard error for 4 replicates per diet.

[b] Reference: no replacement of fish meal (FM) and fish oil (FO).

[c] Replacement of 75% FMFO replaced with *N. oculata* and *Schizochytrium* whole cells

[d] Replacement of 100% of FMFO replaced with *N. oculata* and *Schizochytrium* whole cells

[e] Replacement of 100% of FMFO replaced with *N. oculata*, *Schizochytrium* oil, and canola oil

[f] Saturated fatty acids (SFA) is the sum of all fatty acids without double bonds.

[g] Monounsaturated fatty acids (MUFA) is the sum of all fatty acids with a single bond.

[h] Arachidonic acid (ARA).

i Omega-6 (n-6) Polyunsaturated fatty acids (PUFAs) (sum of all fatty acids with ≥2 double bonds (18:2, 18:3, 20:2, 20:3, 20:4, 22:4, 22:5).

[j] Alpha-linolenic acid (ALA).

[k] Eicosapentaenoic acid (EPA).

[l] Docosahexaenoic acid (DHA).

[m] Total ω3 PUFA

[n] Polyunsaturated fatty acids (PUFA) is the sum of all fatty acids with two or more double bonds.

[o] n-6 long-chain (LC) PUFA (20:2, 20:3, 20:4, 22:4, 22:5).

[p] n-3 long-chain (LC) PUFA (20:3, 20:4, 20:5, 22:5, 22:6).

[q] Ratio calculated for total n-3 PUFA: total n-6 PUFA (n-3/n-6).

Table S2. Macrominerals and trace elements of dietary treatments.

	Diet[a]			
	Reference [b]	NSW75 [c]	NSW100 [d]	NSO100 [e]
Macro minerals (%)				
Phosphorus	1.31	1.09	0.67	0.77
Calcium	3.07	2.06	0.49	0.58
Magnesium	0.15	0.17	0.16	0.18
Potassium	0.02	0.37	0.66	0.75
Sulfur	0.34	0.64	0.92	1.02
Trace elements (mg kg^{-1})				
Copper	164.8	109.6	35.7	39.9
Iron	209.4	263.7	412.0	400.1
Manganese	50.8	43.5	31.6	32.6
Selenium[f]	ND	ND	ND	ND
Zinc	93.27	69.44	211.14	39.18
Arsenic[f]	ND	ND	ND	ND
Boron	25.5	17.4	6.8	9.0
Aluminum	76.5	133.1	243.4	174.3
Mercury	5.7	9.0	10.9	9.9
Lead[f]	ND	ND	ND	ND
Molybdenum[f]	0.12	1.63	1.56	1.67

[a] Mean for two samples per diet.

[b] Reference: no replacement of fish meal (FM) and fish oil (FO).

[c] Replacement of 75% FMFO replaced with *N. oculata* and *Schizochytrium* whole cells

[d] Replacement of 100% of FMFO replaced with *N. oculata* and *Schizochytrium* whole cells

[e] Replacement of 100% of FMFO replaced with *N. oculata*, *Schizochytrium* oil, and canola oil

[f] Not detectable (ND) (<0.00 mg/kg).

Table S3. Total fatty acid profile by percentage of *N. oculata* defatted biomass, *Schizochytrium* sp. whole cells, and *Schizochytrium* DHA oil

	Fatty acid (TFA %)		
	N. oculata defatted biomass	*Schizochytrium* sp. whole cells	Schizochytrium sp. oil
14:0	5.09	9.3	2.12
15:0	0.31	0.5	0.16
16:0	21.31	24.4	25.52
17:0	0.15	ND[a]	0.06
18:0	0.44	0.5	0.85
20:0	0.13	0.1	0.13
22:0	ND	0.1	0.03
24:0	ND	ND	0.00
Total SFA[b]	28.53	9.2	28.94
16:1ω9	6.32	ND	0.00
16:1ω7	23.53	0.2	0.24
18:1ω9	3.28	0.1	0.08
18:1ω7	0.83	ND	0.33
20:1ω9	ND	ND	0.00
20:1ω7	ND	ND	0.00
22:1ω11	ND	ND	0.00
22:1ω9	ND	ND	0.00
24:1ω9	ND	1.4	0.00
Total MUFA[c]	34.34	1.7	0.65
18:2ω6	2.02	ND	0.12
18:3ω6	0.46	0.2	0.05
20:2ω6	ND	ND	0.00
20:3ω6	0.40	0.4	0.13
20:4ω6 ARA[d]	5.82	1.4	0.00
22:4ω6	ND	.1	0.00
22:5ω6	ND	15.8	10.72
Total ω6 PUFA[e]	8.71	17.8	11.01
18:3ω3 ALA[f]	0.08	ND	0.16

18:4ω3	0.00	0.6	0.19
20:3ω3	ND	0.1	0.00
20:4ω3	ND	0.8	0.63
20:5ω3 EPA[g]	27.94	08	0.43
22:5ω3	ND	0.4	0.09
22:6ω3 DHA[h]	ND	43.2	57.90
Total ω3 PUFA[i]	28.03	45.9	59.40
Total PUFA[j]	37.13	63.7	70.41
Total ω6 LC PUFA[k]	6.22	17.6	10.84
Total ω3 LC PUFA[l]	27.94	45.3	59.05
ω3 / ω6 PUFA[m]	3.22	2.58	5.39

[a] Not detectable (ND) (<0.000 ug/g).

[b] Saturated fatty acids (SFA) is the sum of all fatty acids without double bonds.

[c] Monounsaturated fatty acids (MFA) is the sum of all fatty acids with a single bond.

[d] Arachidonic acid (ARA).

[e] Omega-6 (n-6) Polyunsaturated fatty acids (PUFAs) (sum of all fatty acids with ≥2 double bonds (18:2, 18:3, 20:2, 20:3, 20:4, 22:4, 22:5).

[f] Alpha linolenic acid (ALA).

[g] Eicosapentaenoic acid (EPA).

[h] Docosahexaenoic acid (DHA).

[i] Omega-3 (n-3) PUFA (18:3, 18:4, 20:3, 20:4, 20:5, 22:5, 22:6).

[j] Polyunsaturated fatty acids (PUFA) is the sum of all fatty acids with ≥2 double bonds.

[k] n-6 long-chain (LC) PUFA (20:2, 20:3, 20:4, 22:4, 22:5).

[l] n-3 long-chain (LC) PUFA (20:3, 20:4, 20:5, 22:5, 22:6).

[m] Ratio calculated for total n-3 PUFA: total n-6 PUFA (n-3/n-6).

Table S4. Macromineral and trace element composition of *N. oculata* defatted biomass and *Schizochytrium* sp. whole cells, and *Schizochytrium* sp. oil

	N. oculata defatted biomass	*Schizochytrium* sp. whole cells	*Schizochytrium* sp. oil
Macro minerals (%)			
Phosphorus	1.4 ± 0.0	0.92	0.01
Calcium	0.8 ± 0.0	.022	ND[a]
Magnesium	0.6 ± 0.0	0.13	ND
Potassium	1.0 ± 0.0	0.62	0.02
Sulfur	0.5 ± 0.0	2.08	0.04
Trace elements (mg kg^{-1})			
Copper	61.0 ± 3.0	ND	ND
Iron	659.8 ± 14.9	74.37	63.93
Manganese	91.6 ± 2.2	ND	ND
Selenium	1.0 ± 0.0	ND[a]	ND
Zinc	47.0 ± 1.8	0.42	ND
Arsenic	1.0 ± 0.0	0.16	ND
Boron	468.5 ± 53.7	BDL[b]	ND
Aluminum	0.4 ± 0.1	ND	ND
Mercury	0.2 ± 0.0	9.72	9.62
Lead	ND	9.95	10.65
Molybdenum	1.1 ± 0.1	ND	ND

[a] Not detectable (ND) (<0.000 ug/g).

Table S5. Experimental ingredient market prices obtained from Cruz Aquafeed Sustainability Tool (CAST).

Ingredient	Market price [a]	CAST Source
Fish meal (n=30)	1.54 [1.45, 1.63]	Sarker et al. (2020)[18]
Fish oil (n=30)	1.68 [1.64, 1.76]	Sarker et al. (2020)[18]
Canola oil	0.88 [0.86, 0.92]	Sarker et al. (2020)[18]
Co-product meal (raw)	0.44 [0.40, 0.49]	Sarker et al. (2020)[18]
Blood meal (n=9) [b]	0.85 [0.73, 0.93]	USDA (2022a)[g]
Microalgae whole cells	2.38 [1.93, 2.57]	Sarker et al. (2020)[18]
Microalgae oil	4.41 [3.57, 4.76]	Sarker et al. (2020)[18]
Feather meal (n=92) [c]	0.51 [0.46, 0.53]	USDA (2022b)[h]
Corn gluten meal (n=30)	0.62 [0.59, 0.66]	Sarker et al. (2020)[18]
Soy protein concentrate (n=10) [e]	1.30 [1.00, 3.00]	Alibaba (2022)[i]
Wheat gluten meal (n=10) [f]	1.60 [1.28, 2.41]	Alibaba (2022)[i]

[a] Median [and 95% confidence interval] calculated from bootstrap analysis.

[b] Ruminant blood meal or pork blood meal.

[c] Custom report (2010-2019): "Livestock", "Feedstuffs".

[e] Search term: "soy protein concentrate, feed".

[f] Search term: "wheat gluten meal, feed".

[g] USDA, Agricultural marketing service, Feedstuff reports (United States Department of Agriculture, 2022a). Available at: https://www.ams.usda.gov/mnreports/ms_gr850.txt

[h] USDA, Agricultural marketing service, Custom reports (United States Department of Agriculture, 2022b). Available at: https://marketnews.usda.gov/mnp/ls-report-config.

[i] Alibaba, Product Searches, Web accessed March 12, 2022. Available at: Available at: https://www.alibaba.com/.

Table S6. Digestibility of crude protein between dietary treatments.

	Apparent Digestibility Coefficient[ab]				ANOVA	
Nutrient	Reference[c]	NSW75[d]	NSW100[e]	NSO100[f]	F-value	P-value
Crude protein	70.67 ± 4.71[gi]	77.67 ± 1.35[g]	92.69 ± 0.2[h]	91.34 ± 0.75[h]	27.234	<.001

[a] Mean ± standard error for 4 replicates per diet.

[b] Apparent digestibility coefficient (ADC) = $ADC = (1 - ((\%P_{feces} / \%P_{feed}) / (\%Y_2O_{3\,feed} / \%Y_2O_{3\,feces}))) * 100)$

[c] Reference: no replacement of fish meal (FM) and fish oil (FO).

[d] Replacement of 75% FMFO replaced with *N. oculata* and *Schizochytrium* whole cells

[e] Replacement of 100% of FMFO replaced with *N. oculata* and *Schizochytrium* whole cells

[f] Replacement of 100% of FMFO replaced with *N. oculata*, *Schizochytrium* oil, and canola oil

[g h] Mean values not sharing a superscript letter in the same row differ significantly (P < 0.05) from Tukey's HSD test.

[i] One outlier replicate was excluded from this analysis, with only 3 replicates used to calculate the average

Table S7. Amounts of lipid and major omega-3 (n-3) and n-6 polyunsaturated fatty acid (PUFA) of rainbow trout fed dietary treatments for 84 days

Fatty Acid (% TFA)	Whole Body PUFA[a]				ANOVA	
	Reference[b]	NSW75[c]	NSW100[d]	NSO100[e]	*F* Value	*P* Value
18:2n-6 LA [f]	4.19 ± 1.28	4.89 ± 0.72	5.13 ± 0.4	7.43 ± 1.47	1.76	0.208
20:4n-6 ARA [g]	0.11 ± 0.05	0.14 ± 0.02	0.13 ± 0.01	0.14 ± 0.01	0.256	0.855
18:3n-3 ALA [h]	0.23 ± 0.1	0.24 ± 0.05	0.22 ± 0.04	0.49 ± 0.14	2.1	0.154
20:5n-3 EPA [i]	0.38 ± 0.11[k]	0.07 ± 0.01[l]	0 ± 0[l]	0.02 ± 0.02[l]	8.767	0.002
22:6n-3 DHA [j]	0.91 ± 0.2	0.58 ± 0.04	0.55 ± 0.02	0.79 ± 0.06	2.644	0.097

[a] Mean ± standard error for 4 replicates per diet.

[b] Reference: no replacement of fish meal (FM) and fish oil (FO).

[c] Replacement of 75% FMFO replaced with *N. oculata* and *Schizochytrium* flake

[d] Replacement of 100% of FMFO replaced with *N. oculata* and *Schizochytrium* flake

[e] Replacement of 100% of FMFO replaced with *N. oculata*, *Schizochytrium* oil, and canola oil

[f] Linoleic Acid (LA).

[g] Arachidonic acid (ARA).

[h] Alpha linolenic acid (ALA).

[i] Eicosapentaenoic acid (EPA).

[j] Docosahexaenoic acid (DHA).

[k l] Mean values across the row not sharing a common superscript were significantly different as determined by Tukey's HSD test, P<0.05.
